# Identification of Spatiotemporal Associations of Social Determinants of Health on the Incidence of Adverse Birth Outcomes in Louisiana

**DOI:** 10.64898/2026.04.06.26349198

**Published:** 2026-04-07

**Authors:** José Irizarry Ayala, Jian Li, W Susan Cheng, David R Crosslin

## Abstract

**Introduction** Louisiana ranks last in the United States of America in terms of maternal health outcomes. Previous works have highlighted the impact of some social determinants of health on the incidence of adverse birth outcomes. These works have subjectively selected specific social determinants of health from larger datasets. Here, we attempt to replicate their results with objective variable selection techniques. **Methods** By deriving principal components from the Agency of Healthcare Research and Quality's parish-level social determinants of health dataset, we were able to objectively find social determinants of health associations instead of the conventional subjective variable selection approach. Then, we applied Bayesian linear mixed-effects models to calculate more conservative parameter estimates about the effects of social determinants of health on adverse birth outcome incidence. Then, we used local Moran's I to identify clusters of spatially autocorrelated parishes. Finally, we combined the results of these two methods and inspected the relationship between important predictors and clusters of spatial autocorrelation. **Results** We identified several significant effects on the incidence of adverse birth outcomes, including populational composition and economic attainment, and several clusters of high and low incidences of adverse birth outcomes in Louisiana. There was also a concordant relationship between important predictors from our predictive models and the cluster assignments of Local Moran's I. **Conclusion** Our results validate previous works in the subject area and hold implications for precision development of maternal health interventions in Louisiana.

## Full Text Availability

The license terms selected by the author(s) for this preprint version do not permit archiving in PMC. The full text is available from the preprint server.

